# Development of Tissue-Specific Age Predictors Using DNA Methylation Data

**DOI:** 10.3390/genes10110888

**Published:** 2019-11-04

**Authors:** Heeyeon Choi, Soobok Joe, Hojung Nam

**Affiliations:** School of Electrical Engineering and Computer Science, Gwangju Institute of Science of Technology, Gwangju 61005, Korea; heeyeon456@naver.com (H.C.); soobok@gist.ac.kr (S.J.)

**Keywords:** epigenetics, DNA methylation, age prediction, tissue-specific methylation

## Abstract

DNA methylation patterns have been shown to change throughout the normal aging process. Several studies have found epigenetic aging markers using age predictors, but these studies only focused on blood-specific or tissue-common methylation patterns. Here, we constructed nine tissue-specific age prediction models using methylation array data from normal samples. The constructed models predict the chronological age with good performance (mean absolute error of 5.11 years on average) and show better performance in the independent test than previous multi-tissue age predictors. We also compared tissue-common and tissue-specific aging markers and found that they had different characteristics. Firstly, the tissue-common group tended to contain more positive aging markers with methylation values that increased during the aging process, whereas the tissue-specific group tended to contain more negative aging markers. Secondly, many of the tissue-common markers were located in Cytosine-phosphate-Guanine (CpG) island regions, whereas the tissue-specific markers were located in CpG shore regions. Lastly, the tissue-common CpG markers tended to be located in more evolutionarily conserved regions. In conclusion, our prediction models identified CpG markers that capture both tissue-common and tissue-specific characteristics during the aging process.

## 1. Introduction

Aging is often defined as an overall functional decline over time that affects all living organisms [1]. In addition, the human aging process is an important health factor that is still not fully explainable, thus understanding what happens when people age is of great interest [2,3]. In recent years, many cellular and molecular hallmarks of aging have been discovered, including cellular senescence [4], gene expression changes [5,6], and telomere attrition [7,8,9]. Moreover, biological markers of aging involving epigenetic changes have been studied, and DNA methylation has emerged as a promising biomarker of healthy human aging [10]. 

DNA methylation is a representative epigenetic modification that involves the addition of methyl groups to the DNA molecule. DNA methylation is considered to be a crucial epigenetic change because it can alter the activity of genes and is a biomarker that has been implicated in various human diseases, including cancer. Furthermore, many studies revealed that DNA methylation patterns in specific regions have been shown to change along with the aging process [11,12].

As aging-related methylation pattern research has progressed, some studies have identified epigenetic markers that accurately predict the chronological age of healthy people. Bocklandt et al. identified an age predictor using DNA methylation patterns derived from saliva samples [13]. Hannum et al. identified a more accurate age predictor using blood methylation data and reported 71 age-related Cytosine-phosphate-Guanine (CpG) sites known as “Hannum’s clock” [14]. Similarly, a study showed the accurate prediction of age using only three CpG markers in DNA derived from blood [15]. It is important to note that these studies only used DNA from single tissues and primarily focused on blood [16]. On the other hand, Hovarth et al. reported a multi-tissue age predictor that could be applied to all tissues and cell types [17]. This study revealed 353 age-related CpG sites known as “Hovarth’s clock”. Due to its high performance in almost all tissue types, Hovarth’s clock has been widely used as a standard for estimating biological age using reliable epigenetic markers [18,19]. Since the epigenetic clock has emerged, many studies are still underway for methylation-based human aging prediction based on various machine learning techniques. More recently, Li et al. applied a gradient boosting approach to methylation age predictors, which provided even better accuracy for predicting human age [20]. However, these studies did not consider the tissue-specific characteristics of DNA methylation and only revealed CpG sites that explained common aging-related methylation patterns across all tissue types. 

Age-related DNA– methylation shows distinct patterns in different tissues [21]. A comparison of methylation in adipose and liver tissues from young and old mice revealed that the age-related methylation sites in these two tissues showed distinctly different patterns [22]. Furthermore, tissue-common and tissue-specific methylation sites that are significantly correlated with age clearly show different characteristics [23]. These studies indicate a need for new epigenetic age estimators reflecting the specific characteristics of different tissue types.

**Figure 1:**
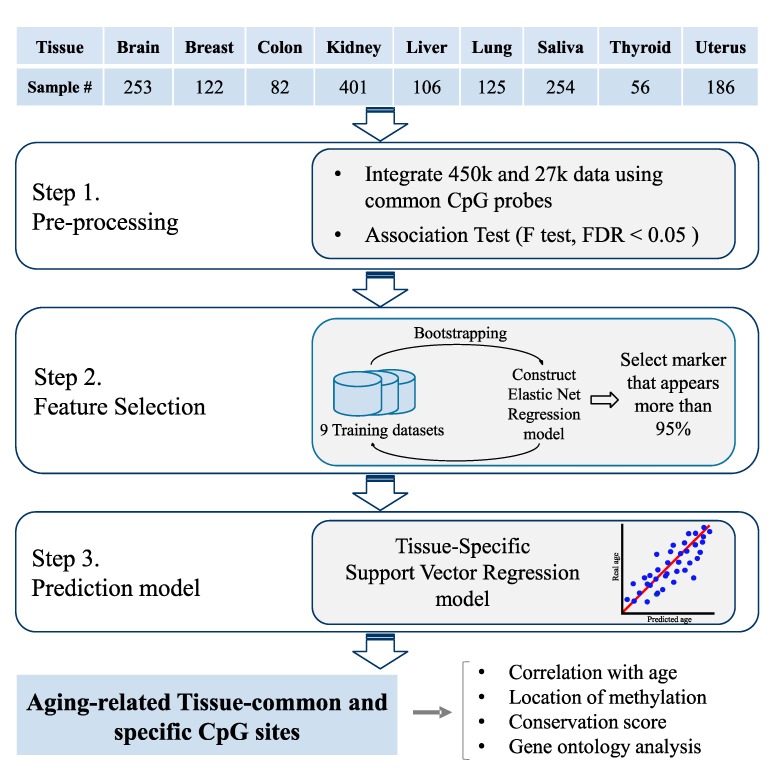
Overview. We collected DNA methylation data from nine healthy human tissues from The Cancer Genome Atlas (TCGA) and Gene Expression Omnibus (GEO) datasets. We used both the 450 K and 27 K platforms to obtain as much data as possible and integrated the two platforms using common Cytosine-phosphate-Guanine (CpG) probes. To select informative features from the high-dimensional array data, initially we filtered the aging-related probes using an F test. Then, we performed feature selection using an elastic net regression and bootstrap analysis. Finally, we constructed tissue-specific support vector regression models for each of the nine tissues. Additionally, we analyzed the selected aging-related features by separating the data into two groups. We compared the characteristics of the CpG probes from the two groups, including the correlation with age, the methylation location, the conservation score, and gene ontology.

Thus, to address the tissue-specific characteristics of DNA methylation changes corresponding to the aging process, in this study, we constructed tissue-specific chronological age predictors using DNA from the same tissues (Figure 1). Here, we hypothesized that DNA methylation pattern may be changed in different manners per each tissue over the aging process. Using DNA methylation data from nine distinct tissues, we constructed tissue-specific age predictors with different epigenetic markers. The validity of the suggested epigenetic markers is tested on the independent test dataset. 

Finally, we identified new aging-related epigenetic markers that exhibit tissue-specific methylation patterns and methylation changes that were common across various tissue types. We further compared the characteristics of tissue-common and tissue-specific aging-related methylation patterns and showed that these patterns clearly differed.

## 2. Materials and Methods 

### 2.1. Tissue-Specific Methylation Datasets

All data were collected from publicly available databases, including The Cancer Genome Atlas (TCGA) [24] and the Gene Expression Omnibus (GEO) [25]. Tissue types with more than 30 normal samples were selected. 

For the training dataset, most of the datasets used were collected from normal tissues in TCGA. The brain dataset consists of cerebellum, frontal lobe, cortex, and pons tissues from 150 subjects [26]. The breast dataset consists of normal tissue from TCGA (Breast invasive carcinoma: BRCA data). The colon dataset consists of adjacent normal tissue from TCGA (Colon adenocarcinoma: COAD an d Rectum adenocarcinoma: READ data). The kidney dataset consists of adjacent normal tissue from TCGA (Kidney renal papillary cell carcinoma: KIRP and Kidney renal clear cell carcinoma: KIRC data). The liver dataset consists of adjacent normal tissue from 62 Taiwanese Hepatocellular carcinoma (HCC) patients [27] and adjacent normal tissue from TCGA (Liver hepatocellular carcinoma: LIHC data). The lung dataset consists of adjacent normal tissue from TCGA (Lung adenocarcinoma: LUAD and Lung squamous cell carcinoma: LUSC data). The saliva dataset consists of samples from Khomani San individuals living in the South African Kalahari [28] and 197 alcohol-dependent subjects [29]. The thyroid dataset consists of adjacent normal tissue from TCGA (Thyroid carcinoma: THCA data). The uterus dataset consists of adjacent normal tissue from TCGA (Uterine Corpus Endometrial Carcinoma: UCEC data) and 152 normal uterine cervix samples [30]. Detailed information is summarized in Table 1.

The independent dataset was derived from normal adjacent tissues from the GEO. The brain dataset consists of 78 samples from the Brodmann area of the cerebral cortex [23]. The breast dataset consists of 23 non-neoplastic breast tissue samples [30] and adjacent normal tissues from patients with invasive breast cancer [31]. The colon dataset consists of 11 normal adjacent colon samples [32] and 87 normal colon biopsies used to study normal aging in the colonic mucosa [33]. The kidney dataset consists of 83 samples of normal adjacent resected kidney tissue [23]. The liver dataset consists of 79 normal liver samples from Germany [34] and control samples from a non-alcoholic fatty liver disease study [35]. The lung dataset consists of normal tissue obtained from smokers from a lung adenocarcinoma study [36]. The saliva dataset consists of saliva samples from an aging study collected from 54 males aged 18 to 73 years [37] and samples from identical twins aged 21 to 55 years [13]. The thyroid dataset consists of normal samples from a methylation study collected from 5 different normal and cancer tissue samples [38]. The uterus dataset was obtained from 152 women involved in a nested prospective case-control study [30]. Detailed information is summarized in Supplementary Table S1.

To generate coherent methylation features, all tissue-specific methylation datasets were selectively collected based on the use of either the Illumina 27 K or Illumina 450 K methylation chip as the assay platform. The Illumina methylation platform is an array-based quantification method for methylation levels at specific loci within the genome. Probes on the 27 K array target regions of the human genome to measure methylation levels at 27,578 CpG dinucleotides in 14,495 genes [39]. The Infinium HumanMethylation450 BeadChip array targets more than 450,000 CpG methylation sites within and outside of CpG islands [40]. The subsequent analyses used CpG features that were commonly measured in both the 27 K and 450 K platforms.

### 2.2. Association Test

To construct the age predictors, CpG probes that commonly appeared in the 27 K and 450 K platforms with less than 10 missing values were used. To combine two different datasets for training, such as in the liver tissue datasets, the 27 K and 450 K datasets were normalized. We applied quantile normalization using the “quantile_transform” function in the preprocessing module provided by “Scikit-learn 0.19.1” [41]. Since more than 20,000 probes were too many to construct the aging models, a linear regression model was constructed for each probe. We performed *F*-tests between the beta values of each CpG probe and age and selected CpG sites with false discovery rate (FDR, Benjamini–Hochberg procedure [42]) values less than 0.05. Here, the *F* value of the regression analysis is the test result of the null hypothesis that the regression coefficients are all zero. Thus, CpG sites that passed the *F*-test (have a significant FDR of less than 0.05) represented CpG features that could have chronological age prediction power [43]. For this *F*-test, we used the “f_regression” function in the feather-selection module provided by “Scikit-learn 0.19.1” [41].

### 2.3. Aging Model Construction

The feature selection process was performed using an elastic net algorithm [44], which is a multivariate linear model implemented in Python 3.5 and the package “Scikit-learn 0.19.1” [41]. The elastic net algorithm uses a penalized regression method that combines the lasso and ridge regression methods. This algorithm is ideal for high-dimensional data where the number of features is greater than the number of samples. We employed a bootstrap analysis by sampling the dataset with replacement and built elastic net regression models for each bootstrap cohort. We repeated this process 500 times and included markers that were present in more than 95% of all bootstrapping processes. Using these selected features, we constructed the final model using a support vector regression (SVR) algorithm. SVR is a regression method that maintains all the main features and characteristics of a support vector machine (SVM) [45]. SVR aims to find a regression function that minimizes the error and has a deviation from the target that is at most ε at all times. To train models using SVR model, we should select several parameters like epsilon, degree of regularization, and kernel functions like linear kernel or RBF kernel. Every optimal parameter was estimated via 10-fold cross-validation by grid search [46,47,48].

### 2.4. Performance Comparison with Multi-Tissue Predictors

To validate the performance of the proposed models, we compared the models with two epigenetic age predictors. The first model is a multi-tissue age predictor that uses 353 CpG markers applicable to each tissue type [17]. For the comparison, we used 3931 methylation samples from 22 tissues and the R function for training described in this study. The second model was a multi-tissue model with SVR. We used the SVR algorithm to predict age using selected features. To validate that the selected features explained the aging process in each tissue, we applied the SVR algorithm in the multi-tissue age predictors. We collected the dataset specified by the study. Using 353 CpG markers as multi-tissue age predictors, we applied the SVR algorithm with the linear kernel. For model construction, parameters such as epsilon and C were selected by 10-fold cross-validation; the same procedure was applied for the tissue-specific age predictors. 

To evaluate model performance, we used three regression metrics. The first metric is a root-mean-square error (RMSE). The RMSE represents the square root of the second sample moment of the differences between predicted and observed values. The second metric is the mean absolute deviation (MAD). The MAD represents the average vertical distance between each point and the Y = X line. The third metric is Pearson’s correlation coefficient, which is also referred to as Pearson’s *r*. The *r* value represents the measure of the linear correlation between predicted and observed values and has a value between +1 and −1, where 1 is a total positive linear correlation, 0 is no linear correlation, and −1 is a total negative linear correlation.

For comparison with the multi-tissue age predictor using other regression metrics, we used two types of metrics. The first metric is the mean absolute percentage error (MAPE). The MAPE is a measure of the prediction accuracy of a forecasting method in statistics. The absolute value in this calculation is summed for every forecasted point in time and divided by the number of fitted points n. Multiplying by 100% makes the value a percentage error [49]. The other metric is Theil’s U statistic. This metric is a relatively accurate measure that compares the forecasted results with the results of forecasting with minimal historical data [50].

### 2.5. Conservation Score Calculation

We examined the phastCons score of the CpG sites within the genomic regions provided by the human methylation 450 K annotation file [51]. PhastCons score is a probability that each nucleotide belongs to a conserved element through the evolution process. In this case, we used Phastcons score for multi alignment fo 99 vertebrate genomes to the human genome. We calculated the average phastCons score for each base of the genomic regions.

### 2.6. GO Analysis

The GO analysis was performed using the ClueGO plugin in Cytoscape 3.5.1 [52]. We used the GO biological process database as a reference. Gene lists were selected using the closest gene to each CpG marker that was provided by the Illumina human methylation 27 K platform annotation file. 

## 3. Results

### 3.1. Tissue-Specific Age Prediction Model

In this study, we used 21,396 probes that could be measured using the Illumina Infinium II assay and were common to both the 27 K and 450 K platforms. 

Initially, the number of CpG features was more than 100 times greater than the average number of samples. Therefore, to filter out features that are not related to aging, we performed association tests between the β-values of each CpG site and age. As a result, we selected CpG sites that had a false discovery rate (FDR) < 0.05. After the association test, the average number of features of each tissue was reduced to 8800. Then, we selected features to be used in the age prediction model by using a penalized multivariate regression method (Table 2). Additionally, we counted the number of occurrence in features from each tissue-specific age predictors. cg22736354 methylation marker is found as common marker of all nine tissue-specific age predictors. Detailed information is provided in Supplementary Table S3. Finally, using these selected features, we constructed nine tissue-specific age prediction models using a SVR algorithm (Figure 1). We used the SVR algorithm in the final regression modeling because it showed lower mean absolute deviation (MAD) values than the elastic net regression in almost all tissue types. The results are presented in Supplementary Figure S1. In addition, the SVR method had better epigenetic age prediction performance than the multivariate regression method [53]. In previous epigenetic aging studies, elastic net regressions have been typically used for both feature selection and age prediction [14,15,16,17]. As methylation array data are not abundant in normal samples, use of the SVR algorithm instead of a simple multivariate linear regression appeared to perform well in analyses of the high-dimensional bioinformatic data.

**Figure 2:**
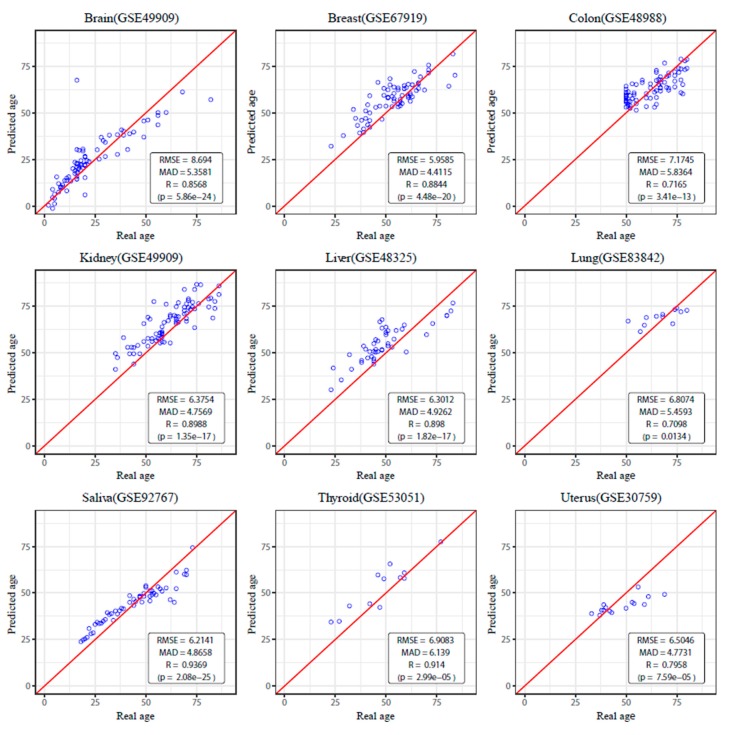
External Validation. We tested the tissue-specific age predictors listed in an independent dataset and found that the mean absolute deviation (MAD) was 5.119 years and the correlation coefficient was 0.845 on average for the nine models. The results in the brain (MAD = 5.358, *r* = 0.859), breast (MAD = 4.412, *r* = 0.884), colon (MAD=5.836, *r* = 0.717), kidney (MAD = 4.757, R=0.899), liver (MAD = 4.926, *R* = 0.898), lung (MAD = 5.459, *R* = 0.710), saliva (MAD = 4.866, *R* = 0.937), thyroid (MAD = 6.14, *R* = 0.910), and uterus (MAD = 4.773, *R* = 0.796) are shown.

We validated the performance of the tissue-specific age predictors using several metrics, including the root mean squared error (RMSE), mean absolute deviation (MAD), and Pearson’s correlation coefficient (*r*), to compare the predicted age to the chronological age. According to these accuracy measures, the tissue-specific age predictors performed well in all nine tissue types. We performed 10-fold cross-validation and external valiation to verify that the model was well trained. *K*-fold cross-validation is a model validation technique that uses a partitioned original sample to test a model trained with the remaining *k*-1 subsamples. We set the parameter k as 10 [47]. For the 10-fold cross-validation, the RMSE was 4.679 (years), MAD was 3.519 (years), and *r* was 0.9619 on average for the nine tissue models (Supplementary Figure S2). To test that nine tissue-specific age predictors performed well in real-world methylation data, we also validated the model in an independent test dataset for every nine tissues from GEO database. Regarding the external validation, the RMSE was 6.512 (years), MAD was 5.119 (years), and *r* was 0.845 on average (Figure 2).

### 3.2. Comparison with the Multi-Tissue Age Predictor

**Figure 3:**
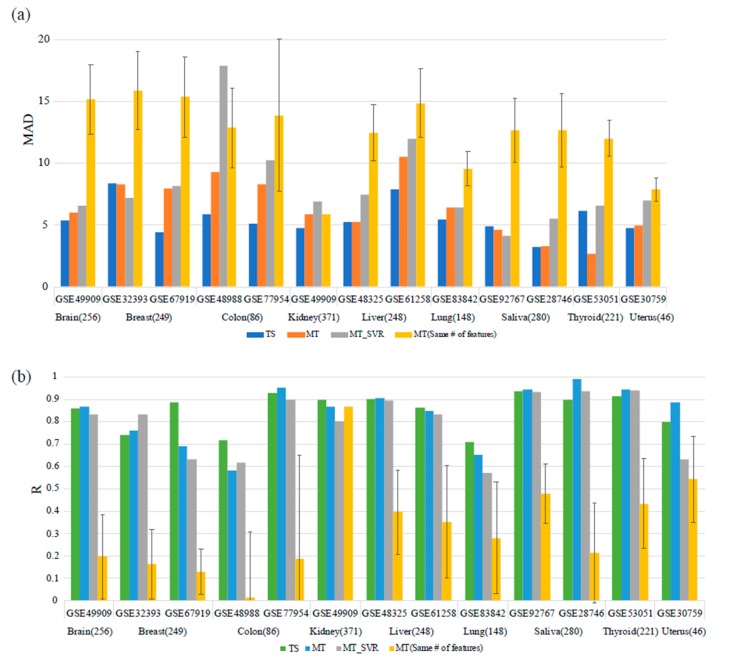
Comparison with a previous study. The results were compared between the proposed tissue-specific age predictors and multi-tissue age predictors for an independent dataset collected in this study. For the abbreviations, TS means Tissue-specific age predictor we suggested, MT means Multi-tissue age predictor and MT_SVR is Support Vector Regression Model constructed by using features of multi-tissue age predictor for equitable comparison. MT(same # of features) means a Multi-tissue age predictor using the same number of features in TS model. The numbers in parentheses refer to the number of markers of each tissue-specific model. (**a**) When comparing MAD values, the tissue-specific age predictors showed better performance except for the breast and thyroid tissue datasets. (**b**) When comparing the *R* values as coefficient of correlation, some tissues showed better performance in the multi-tissue age predictors, but the results were similar.

To validate the tissue-specific age predictors, we compared the performance of two models. The first model was the multi-tissue age prediction model described by Hovarth, which used 353 aging-related CpG markers [17] The second model was a multi-tissue age predictor that applied the SVR algorithm. In our results, generally, the tissue-specific age predictors had lower MAD values than the multi-tissue age predictors (Figure 3). For comparison, we used the same test dataset that we used for validating the proposed age predictors (see Methods 2.1 for details). Also these test datatsets were not used in the training process of multi-tissue age predictor, which were also appropriate for comparing the performance of multi-tissue age predictor(Supplementary Table S2).

Although the r values of the multi-tissue age predictors were slightly higher than those of the tissue-specific age predictors in some tissues, the models exhibited similar results. We compared the models using other metrics that are commonly used in regression problems, such as the mean absolute percentage error (MAPE) and Theil’s U statistics, and found that the tissue-specific age predictors tended to show better results (Supplementary Figure S3). As these improved results may be due to the superiority of SVR and not the explanatory power of the selected features, we applied the SVR algorithm to the dataset and features described by Hovarth. As a result, the proposed tissue-specific age predictors generally showed better performance than the multi-tissue age predictors with the SVR model. This result shows that tissue-specific age predictors can reflect the aging process well despite the smaller numbers of training data and features. For some dataset like saliva (GSE92676), or thyroid (GSE53051) multi-tissue age predictor showed better results in prediction error. However, tissue-specific age predictors showed better or comparable results using a smaller number of training samples and features. Considering that multi-tissue age predictors used about 4000 samples for training the model, which also added plentiful blood dataset samples, tissue-specific age predictors used only about 200 samples for each tissue on average for training and used a smaller number of features to predict the age of the samples. Despite the unfair condition, tissue-specific age predictors predicted age of samples with comparable level with multi-tissue age predictors by also considering the tissue-specific properties. We also compared how many selected features overlap with Hovarth’s clock (353 CpGs). Methylation sites used for features of multi-tissue age predictors more belonged to tissue-common group (Supplementary Figure S4).

In addition, our results suggest that age-related DNA methylation changes have both tissue-specific and tissue-common patterns, and we have selected the features that reflect both of these characteristics.

To determine whether the tissue-common and tissue-specific patterns had different characteristics, we divided the selected features into two groups and analyzed their features. We defined the “tissue-common group” as CpG sites that were commonly used in more than two tissue models. In contrast, the “tissue-specific group” was defined as CpG sites that were uniquely used in only one tissue model. In total, we found 247 tissue-common aging markers and 1,213 tissue-specific aging markers. The number of aging markers in each tissue type is listed in Table 2. 

Several studies have shown aging-related methylation patterns in blood. Thus, we aimed to validate that the tissue-common group was consistent with the findings from these previous studies. In a review on DNA methylation and healthy human aging, the authors filtered 11 CpG sites associated with age in the blood that appeared in at least four of the eight studies [54]. Nine of the 11 CpG sites in our derived tissue-common group were congruent with aging. Furthermore, compared with Hovarth’s clock, 353 CpG sites were included in more of the tissue-common features than in the tissue-specific features. These results show that the tissue-common markers in our models reflect epigenetic aging patterns that generally appear throughout various tissues.

### 3.3. Tissue-Common and Tissue-Specific Features of Age-Dependent Methylation in Tissues

**Figure 4:**
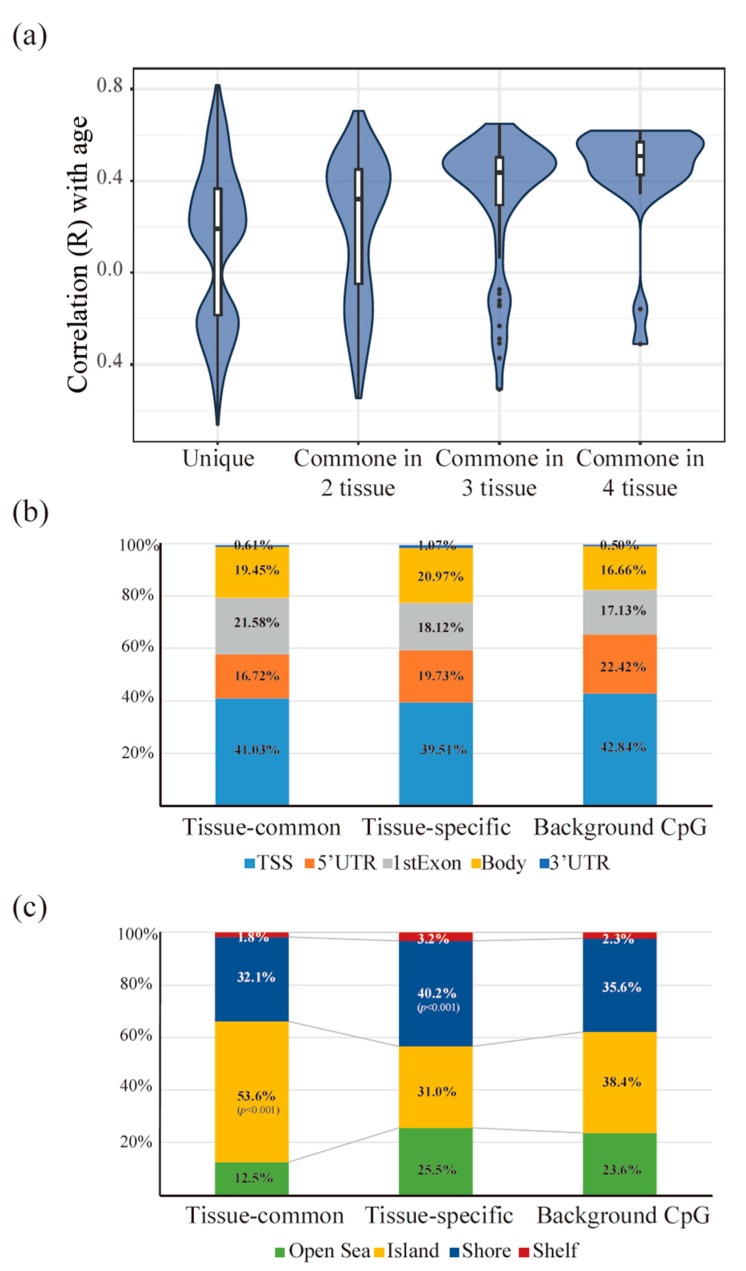
Different patterns between the tissue-common and tissue-specific groups. Different characteristics of the methylation direction and location between the tissue-common and tissue-specific groups (**a**–**c**) (**a**) When the correlation coefficient of the beta values of each methylation site and age were compared, more positive ageCGs were found in aging-related CpG sites. However, the ratio of negative ageCGs was higher in the unique groups defined as tissue-specific. In addition, the ratio of positive ageCGs increased as the number of appearances in the tissue model increased. (**b**) When the methylation locations based on the gene structure were compared, no significant changes were found between the two groups (*p* = 0.4059). We compared the methylation location sites in the 27 K platform and found more gene body regions that contained aging-related CpG sites (*p* < 10^−6^). (**c**) We compared the locations of the methylation sites in the CpG islands and CpG shores and found more CpG island regions in the tissue-common group (*p* < 10^−6^) and more CpG shore regions in the tissue-specific group (*p* = 0.0001).

We hypothesized that tissue-specific aging signatures would show methylation patterns that differed from those commonly observed. To validate this assumption, we compared the tissue-common methylation patterns with the tissue-specific methylation patterns. First, we compared the direction of methylation that accompanied the aging process. We defined “positive ageCGs” as CpG sites whose methylation increased during the aging process and “negative ageCGs” as CpG sites whose methylation decreased during the aging process. Generally, more positive ageCGs were found, but the tissue-specific group had more negative ageCGs than the tissue-common group (Supplementary Figure S5). Interestingly, as the number of markers that appeared in the tissue types increased, the ratio of positive ageCGs also increased (Figure 4a). This finding could suggest that general methylation patterns increase with age; however, decreasing methylation patterns that were associated with tissue-specific aging were also observed. 

Also, to compare the general methylation patterns that emerged during aging, we divided the data into a younger group and an older group. For the training data, we selected the five oldest and five youngest samples from each tissue type. For each tissue, the age difference between the two groups was greater than 40 years. No significant difference was observed in the total 27 K methylation patterns between the two groups. However, by comparing the methylation patterns of the selected aging-related features, we observed that the older group tended to be more highly methylated than the younger group in each tissue type except for the lung tissue (Supplementary Figure S6). This result is consistent with the abundance of positive ageCGs found in aging-related methylation sites. 

Next, we compared the ratio of the gene structures in which each CpG marker was located. The distribution of the locations in the gene structures in both the common and unique CpG groups did not significantly differ (Fisher’s exact test, *p* = 0.4059). However, the aging-related methylation markers were more likely to be located in the gene bodies than the location distribution of all CpG sites in the 27 K platform (Fisher’s exact test, *p* < 10^−6^) (Figure 4b). Although the result was not significant, the tissue-common group included more exon regions than the tissue-specific group (Fisher’s exact test, *p* = 0.1303) (Supplementary Figure S7). In addition, we compared the ratios of the CpG islands to the CpG shore regions in which each CpG marker was located. The tissue-common group tended to be located in the CpG island regions, whereas the tissue-specific group tended to be located in the CpG shore regions (Fisher’s exact test, *p* < 10^−6^, *p* = 0.0001) (Figure 4c). These results are consistent with results from a previous study comparing age-related tissue-common and tissue-specific patterns in four tissues [23]. The results show that tissue-specific and tissue-common methylation patterns appear in different locations and that each tissue type may have distinct mechanisms that operate during the normal aging process. 

**Figure 5:**
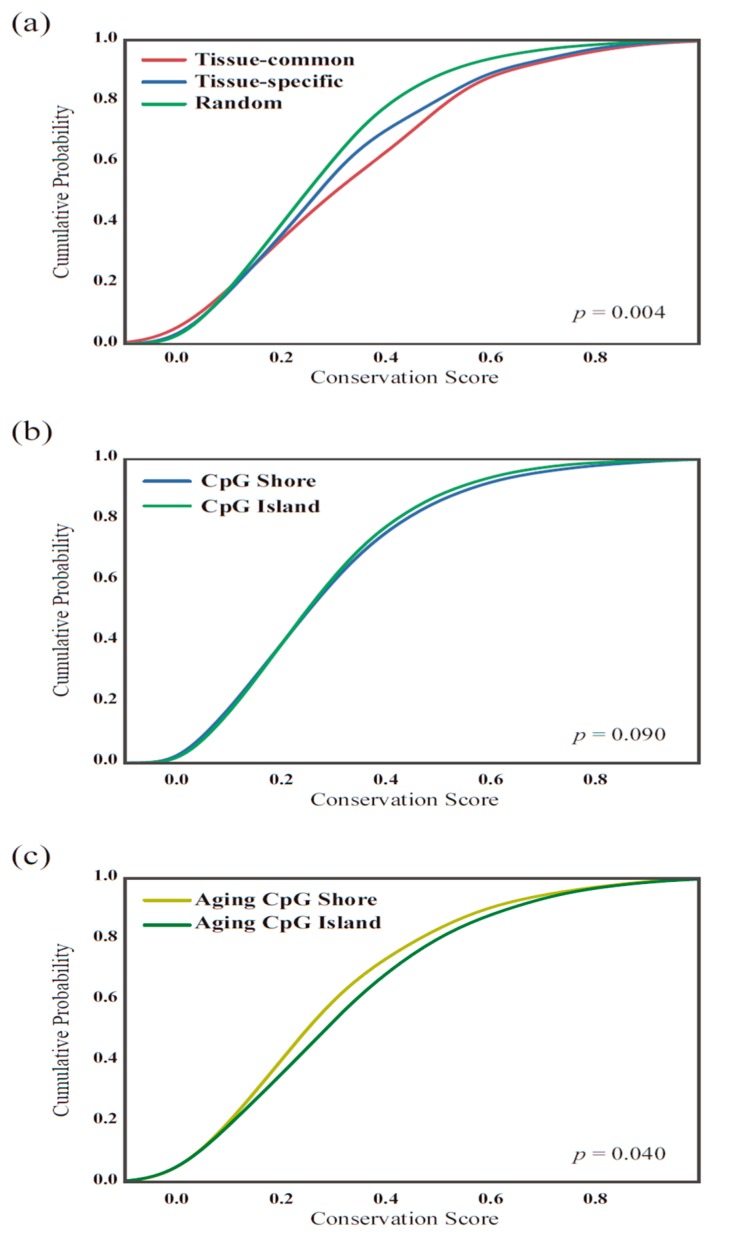
Conservation scores. (**a**) Comparison of the cumulative distribution of the average conservation scores between the tissue-common and tissue-specific groups. The two distributions of the tissue-common and tissue-specific group show significant differences using the Kolmogorov-Smirnov test (*p* = 0.004). (**b**) As the two groups showed differences in the methylation locations based on CpG islands, we compared the conservation score of each CpG site in the CpG island and shore regions from the 27 K methylation platform but did not find a significant difference (*p* = 0.09). (**c**) However, the conservation scores of the aging-related CpG island and shore regions slightly differed (*p* = 0.04).

Furthermore, we assumed that the methylation patterns appearing in various tissues might be more evolutionarily conserved than the patterns from tissue-specific mechanisms. To test this assumption, we calculated the average evolutionary conservation (phastCons) score of the chromosome region of each CpG marker from the Illumina annotation file (see Methods for details). As expected, the distribution of the conservation scores of the tissue-common and tissue-unique groups differed (Kolmogorov-Smirnov test, *p* = 0.00413). The aging-related CpG markers tended to be located in more conserved regions than randomly selected CpG sites (Figure 5a). This result may be due to the original conservation score differences in the CpG island regions. Thus, we compared the conservation score distributions of the whole CpG island and shore regions provided by the Illumina 27 K annotation file but found few differences (*p* = 0.09) (Figure 5b). However, when we compared the conservation score distributions of the CpG islands and shore regions of the aging markers, we found significant differences (*p* = 0.04) (Figure 5c). We thought that this conservation score difference was not due to the known evolutionary conserveness difference in CpG Island and Shore region. However, it is because of tissue-specific and common methylation differences throughout the aging process.

### 3.4. The Functionality of Tissue-Common and Specific Methylation Regions

Finally, we performed a gene ontology (GO) analysis to validate the function of the closest gene to the selected markers. We assumed that the selected methylation markers affected the function of the gene closest to the site. To analyze the gene ontology, the markers were divided into the positive ageCG and negative ageCG groups, since we suspected that the functions of the positive ageCGs and negative ageCGs might differ.

**Figure 6:**
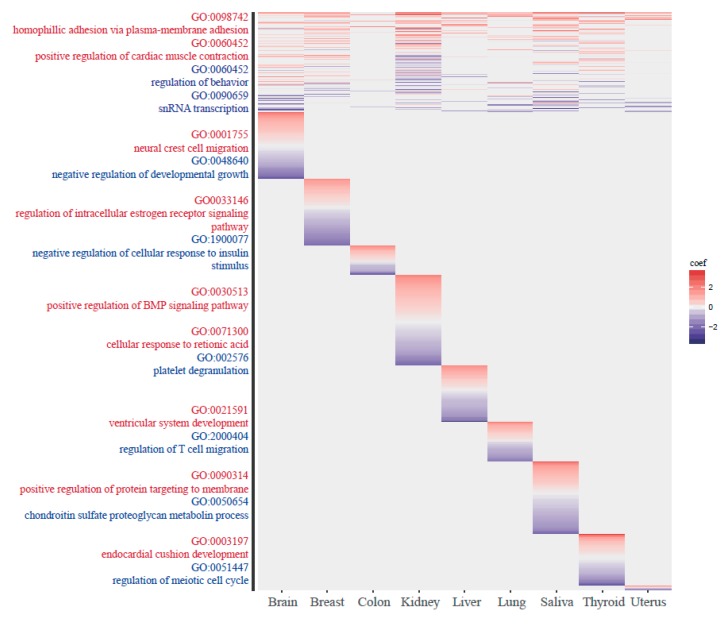
Gene ontology (GO) analysis. GO terms for the closest gene of each methylation group are shown. Because we conducted the gene ontology analysis by separating the sites into positive and negative ageCGs, red color indicates the GO terms from the positive ageCGs and blue color indicates the negative ageCGs.

In the tissue-common group, GO terms such as cell-cell adhesion and cardiac muscle contraction appeared in the positive direction, and GO terms such as regulation of behavior and snRNA transcription appeared in the negative direction. In the tissue-specific group with positive ageCGs, tissue-specific GO terms were associated with brain and breast tissue, including neural crest cell migration and mammary gland lobule development. In the tissue-specific group with negative ageCGs, immune-related terms were found, such as regulation of T cell migration. The number of T cells and the distribution of lymphocyte subsets have been shown to vary with age [55]. The GO terms for each group are shown in Figure 6, and the top lists of GO terms for tissue-common and tissue-specific group are provided in Supplementary Tables S4 and S5.

## 4. Discussion

Although many studies have investigated aging-related epigenetic markers using methylation arrays, these studies are limited by only considering the common characteristics of aging across tissues. In this study, we constructed nine tissue-specific age prediction models to study the tissue specificity of DNA methylation. Using a penalized regression model, reliable features that affect aging were selected. We selected fewer than 400 epigenetic markers for each tissue that may reflect the aging processes of that tissue. The number of selected features differed for each tissue, and these differences may reflect the different aging processes of each tissue. For instance, tissue models with more CpG features may suggest that the tissues have undergone more biological changes during the aging process. However, these differences could also be the result of the different numbers of features derived from the model training process. 

Generally, the tissue-specific age predictors showed better performance than the multi-tissue predictors despite the smaller number of features and the smaller training dataset. However, obtaining better results in the comparison does not necessarily mean that the tissue-specific age predictors represent a better biological age prediction method. The multi-tissue age predictors estimated the chronological age of each tissue with high accuracy. However, this model was limited because the specificity of DNA methylation patterns in different tissues was not considered. Moreover, tissue-specific age predictors have an advantage in providing tissue-specific biomarkers. By constructing a model for each tissue type, we found different aging markers that did not appear in other tissues. This method was limited because the feature selection was time-consuming. Moreover, because this method was applied to only nine tissues, finding aging-related methylation markers in other tissues could prove to be difficult. 

Although the two models have pros and cons, the better performance of the tissue-specific age predictors suggests that aging-related DNA methylation patterns have both tissue-specific and tissue-common characteristics. Therefore, we compared the tissue-common and tissue-specific aging-related DNA methylation patterns to determine whether the two groups showed different characteristics. First, more positive ageCGs were positively correlated with age, suggesting that generally aging-related methylation regions increased throughout an individual’s lifetime. Interestingly, the tissue-specific groups had more negative ageCGs than positive ageCGs; this phenomenon showed that decreasing methylation patterns also existed in tissue-specific aging mechanisms. In addition, these two groups are located in remarkably different regions based on their proximity to CpG islands. The tissue-common methylation regions involved in aging tended to be more conserved than the tissue-specific regions. This result suggests that the tissue-specific and tissue-common groups show different patterns because biologically important regions are more conserved throughout evolution. 

Finally, the two groups showed different functionalities. To determine the differences in each methylation group, we performed a GO analysis by separating the data into positive and negative ageCGs. Interestingly, the genes in regions of both positive and negative tissue-common groups tended to show GO terms such as muscle contraction and behavior. Furthermore, each tissue showed different functions, although not all tissues showed the same tissue-specific ontology terms. Although tissue-specific terms typically appeared in the negative ageCGs, tissue-specific terms in the positive ageCGs were found from brain and breast tissue. We believe that this finding is the result of the small number of negative ageCGs; thus, many tissue-specific terms were not statistically significant. In addition, determining which tissue is aging at the fastest rate is an important question in aging studies. Some studies have investigated different aging rates in specific tissues [56,57]. However, comparing different biological ages predicted by age predictors of each tissue is inappropriate, because the prediction may be the result of the bad performance of the model. We did not directly address the rate of aging between tissues. We investigated that the degrees of the slope between age and methylation level of cg22736354, which is one of the tissue-common markers. According to the aging process, we noted that the slopes of the methylation level of this single cg22736354 were different for each tissue type. We confirmed that the slope of colon tissue is smallest, and in the brain tissue, the slope value is higher than other tissues. These results suggest that aging marker in colon tissue more slowly than other tissues (supplementary Table S6).

## 5. Conclusions

This study revealed epigenetic age predictors that reflect the characteristics of DNA methylation and tissue-specific aging mechanisms. The tissue-common markers found in this study may be used as reliable epigenetic aging markers that reflect aging-related DNA methylation changes that commonly appear in many tissues. Furthermore, these tissue-specific markers may be used as targets to identify tissue-specific epigenetic aging signatures. However, the cellular mechanisms of DNA methylation require further investigation. Since a clear description of the relationship between DNA methylation and aging is lacking, each tissue type may have different biological mechanisms, and thus, both tissue-common and tissue-specific aging mechanisms need to be considered in future studies. 

Although the proposed tissue-specific age-prediction model showed remarkable performance, the method still has room for improvement. First, with the development of next-generation sequencing (NGS) technology, non-CpG methylation has been measured and observed, and the role of non-CpG methylation in specific cells has been interpreted to some extent in recent years. In this sense, non-CpG methylation can also be considered an aging-prediction marker [58]. Additionally, with the accumulation of datasets, more sophisticated machine learning models [59,60,61], such as deep neural networks (DNN) [62,63], can be applied to identify and interpret age-related features that cannot be captured with simple linear or non-linear regression models.

## Figures and Tables

**Table 1:** Training dataset. To train the model, we used DNA methylation data from healthy or normal adjacent samples obtained from nine different tissues. We collected methylation array data obtained using both the 27 K and 450 K platforms from TCGA and GEO datasets.

Data Set	Tissue Type	The Number of Patients		Age Range	Platform
GSE15745 [26]	Brain	253		16–95	HumanMethylation 27 K
TCGA [24]	Breast	95	122	28–90	HumanMethylation 450 K
		27		35–88	HumanMethylation 27 K
TCGA [24]	Colon	45	82	40–90	HumanMethylation 450 K
		37		43–90	HumanMethylation 27 K
TCGA [24]	Kidney	205	401	31–90	HumanMethylation 450 K
		196		33–86	HumanMethylation 27 K
TCGA [24]	Liver	49	106	20–81	HumanMethylation 450 K
GSE37988 [27]		57		20–79	HumanMethylation 27 K
TCGA [24]	Lung	74	125	40–86	HumanMethylation 450 K
		51		51–83	HumanMethylation 27 K
GSE99029 [28]	Saliva	57	254	21–91	HumanMethylation 27 K
GSE34035 [29]		197		21–55	HumanMethylation 27 K
TCGA [24]	Thyroid	56	56	15–81	HumanMethylation 450 K
TCGA [24]	Uterus	34	186	36–90	HumanMethylation 450 K
GSE30758 [30]		152		19–55	HumanMethylation 27 K

**Table 2:** The number of features of each tissue. This table lists the number of final features used for model construction. We used these features for the subsequent feature analysis.

	Brain	Breast	Colon	Kidney	Liver	Lung	Saliva	Thyroid	Uterus	Total
Total	256	249	86	371	248	148	280	221	46	1460
Common	93	92	17	153	53	49	103	99	35	247
Specific	163	157	69	218	195	99	177	122	11	1213
Positive	170	202	54	281	186	67	232	151	35	989
Negative	86	47	32	90	62	81	48	70	11	471

## Supplementary Materials

The following are available online at https://www.mdpi.com/2073-4425/10/11/888/s1, Figure S1: Comparison of SVR and Elastic Net regression method; Figure S2: Internal validation; Figure S3: Comparison with other metric Comparison with other metric; Figure S4: Number of common features with hovarth; Figure S5: Ratio of positive and negative ageCGS; Figure S6a: General methylation pattern difference in young and old group; Figure S6b: Aging-related methylation pattern difference in young and old group; Figure S7: Exon and intron ratio in tissue-common and tissue-specific group, Table S1: Independent dataset; Table S2: Common dataset with Hovarth et al; Table S3: Number of Common features in tissue-common CpG groups; Table S4: Gene Ontology Analysis of tissue-common group; Table S5a: Gene Ontology Analysis of positive ageCGs of tissue-specific group; Table S5b: Gene Ontology Analysis of negative ageCGs of tissue-specific group; Table S6: The comparison with cg22736354 methylation and age in each tissue, Supplementary_Aging_marker_list.xlsx contains detailed CpG marker information.
