# Supplementary material for: Development of Tissue-Specific Age Predictors Using DNA Methylation Data
Source: Genes (Basel). 2019 Nov 4;10(11):888. doi: 10.3390/genes10110888 (PMC6896025; doi:10.3390/genes10110888)
Supplement: Supplementary file 1 [file genes-10-00888-s001.zip › Supplymentary_20191101.pdf]

genes

Development of tissue-specific age predictors using DNA methylation data

Heeyeon Choi, Soobok, Joe, Hojung Nam*

School of Electrical Engineering and Computer Science, Gwangju Institute of Science of Technology, Gwangju, 61005, Republic of Korea

Data Set	Tissue Type	# of patients		Age range	Platform
GSE49909	Brain	78		1-82	HumanMethylation 27
GSE32393[2]	Breast	22	91	19-75	HumanMethylation 450
GSE67919[3]		69		23-84	HumanMethylation 450
GSE77954[4]	Colon	11	98	49-85	HumanMethylation 450
GSE48988[5]		87		50-80	HumanMethylation 450
GSE49909[1]	Kidney	82		2-86	HumanMethylation 27
GSE61258[6]	Liver	51	96	33-86	HumanMethylation 450
GSE48325[7]		45		23-83	HumanMethylation 450
GSE83842[8]	Lung	11		51-80	HumanMethylation 450
GSE92767[9]	Saliva	53	99	18-73	HumanMethylation 27
GSE28746[10]		36		21-55	HumanMethylation 27
GSE53051[11]	Thyroid	12		23-77	HumanMethylation 450
GSE30579[2]	Uterus	15		33-69	HumanMethylation 27

Supplementary Table 1. Independent dataset. For validating tissue-specific age predictors, we collected independent dataset of every nine tissues from GEO database. This table shows the specific information about the dataset that we used.

GSE49909((Day, Waite et al. 2013), GSE32393(Zhuang, Jones et al. 2012), GSE67919(Hair, Xu et al. 2015), GSE77954(Qu, Sandmann et al. 2016), GSE48988(Noreen, Röösli et al. 2014), GSE61258(Horvath, Erhart et al. 2014), GSE48325(Ahrens, Ammerpohl et al. 2013), GSE83842(Kajiura, Masuda et al. 2017), GSE92767(Hong, Jung et al. 2017), GSE28946(Bocklandt, Lin et al. 2011), GSE53051(Timp, Bravo et al. 2014)

Usage	Dataset	Tissue type	Overlapped in MT predictor (Horvath 2013)
Train	GSE15745	Brain	X
	TCGA(BRCA)	Breast	O (test)
	TCGA(COAD,READ)	Colon	O(test)
	TCGA(KIRP, KIRC)	Kidney	O (train)
	TCGA(LIHC)	Liver	O (test)
	GSE37988		O (train)
	TCGA(LUAD, LUSC)	Lung	O (train)
	GSE99029	Saliva	X
	GSE34035		O (train)
	TCGA(THCA)	Thyroid	O (train)
	TCGA(UCEG)	Uterus	O (test)
	GSE30758		O (test)
Test	GSE49909	Brain	X
	GSE32393	Breast	O (train)
	GSE67919		X
	GSE77954	Colon	X
	GSE48988		X
	GSE49909	Kidney	X
	GSE61258	Liver	X
	GSE48325		X
	GSE83842	Lung	X
	GSE92767	Saliva	X
	GSE28746		X
	GSE53051	Thyroid	X
	GSE3057	Uterus	X

Supplementary Table 2. Common dataset with Hovarth et al. There are some common training dataset with dataset used in Hovarth et al. However, For independent dataset for validating the model performance, we almostly used the dataset not used in the process of training the multi-tissue age predicto

Number of common features	Count	CpG name
9	1	cg22736354'
8	1	cg25148589'
7	4	cg06458239', 'cg10523019', 'cg04084157', 'cg12373771'
6	5	cg06493994', 'cg04528819', 'cg22809047', 'cg22719623', 'cg20300246'
5	12	cg03664992', 'cg20692569', 'cg06144905', 'cg19945840', 'cg00548268', 'cg02681442', 'cg18236477', 'cg27320127', 'cg00059225', 'cg25942450', 'cg19560758', 'cg12422450'
4	25	cg05266781', 'cg19996355', 'cg15747595', 'cg17758721', 'cg06291867', 'cg06156376', 'cg26005082', 'cg21589115', 'cg22909609', 'cg08209133', 'cg17965019', 'cg03679581', 'cg10947146', 'cg00107187', 'cg02654291', 'cg12078929', 'cg10235817', 'cg21296230', 'cg00055233', 'cg10608333', 'cg09212468', 'cg17497271', 'cg06646021', 'cg14319409', 'cg12402251'
3	55	cg23854009', 'cg27524460', 'cg27553955', 'cg00563932', 'cg06493386', 'cg12073594', 'cg18809289', 'cg23941599', 'cg09809672', 'cg08536841', 'cg09816471', 'cg23124451', 'cg22395019', 'cg18008766', 'cg18943383', 'cg12024906', 'cg07922606', 'cg13854874', 'cg06121469', 'cg07408456', 'cg26219051' (Continue at Supplementary Aging marker list.xlsx)
2	142	cg04527918', 'cg12261786', 'cg19728223', 'cg03975694', 'cg16483916', 'cg08260959', 'cg26381783', 'cg19586576', 'cg23855989', 'cg25589890', 'cg17471102', 'cg25538571', 'cg25426302', 'cg12554573', 'cg03330678', 'cg02228185', 'cg05590982', 'cg07684796', 'cg16584172', 'cg16488098', 'cg25136310', (Continue at Supplementary Aging marker list.xlsx)

Supplementary Table 3. Number of Common features in tissue-common CpG groups. This table shows the number of appearance in tissue-common aging markers in each tissue groups. More detailed information about markers are listed in Supplementary feature list file.

r.

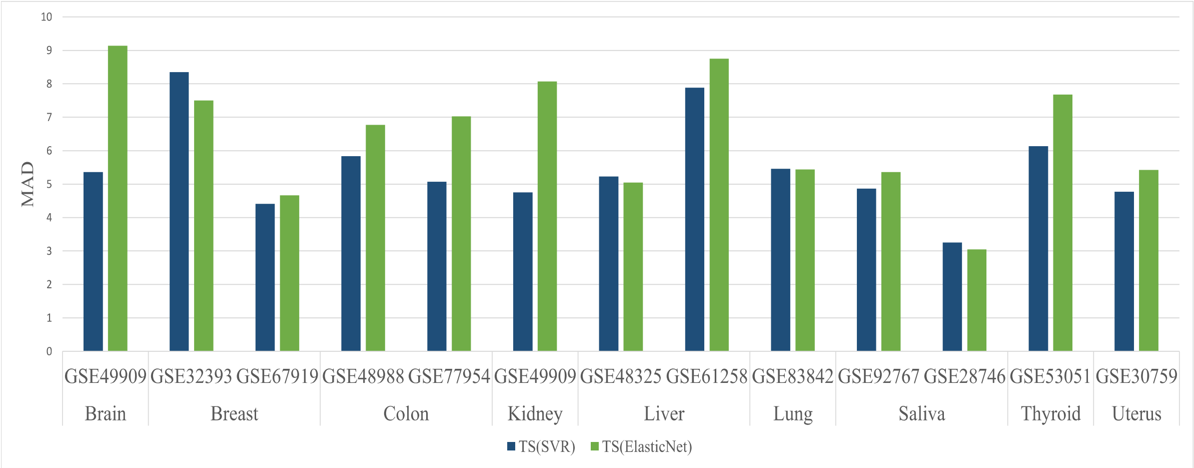

Supplementary Figure 1. Comparison of SVR and Elastic Net regression method. Using selected features, we applied SVR and Elastic Net regression algorithm. When we compared the Mean Absolute Deviation of two models, the model applied SVR algorithm showed better performance in every independent dataset. Thus we selected SVR algorithm for final model construction.

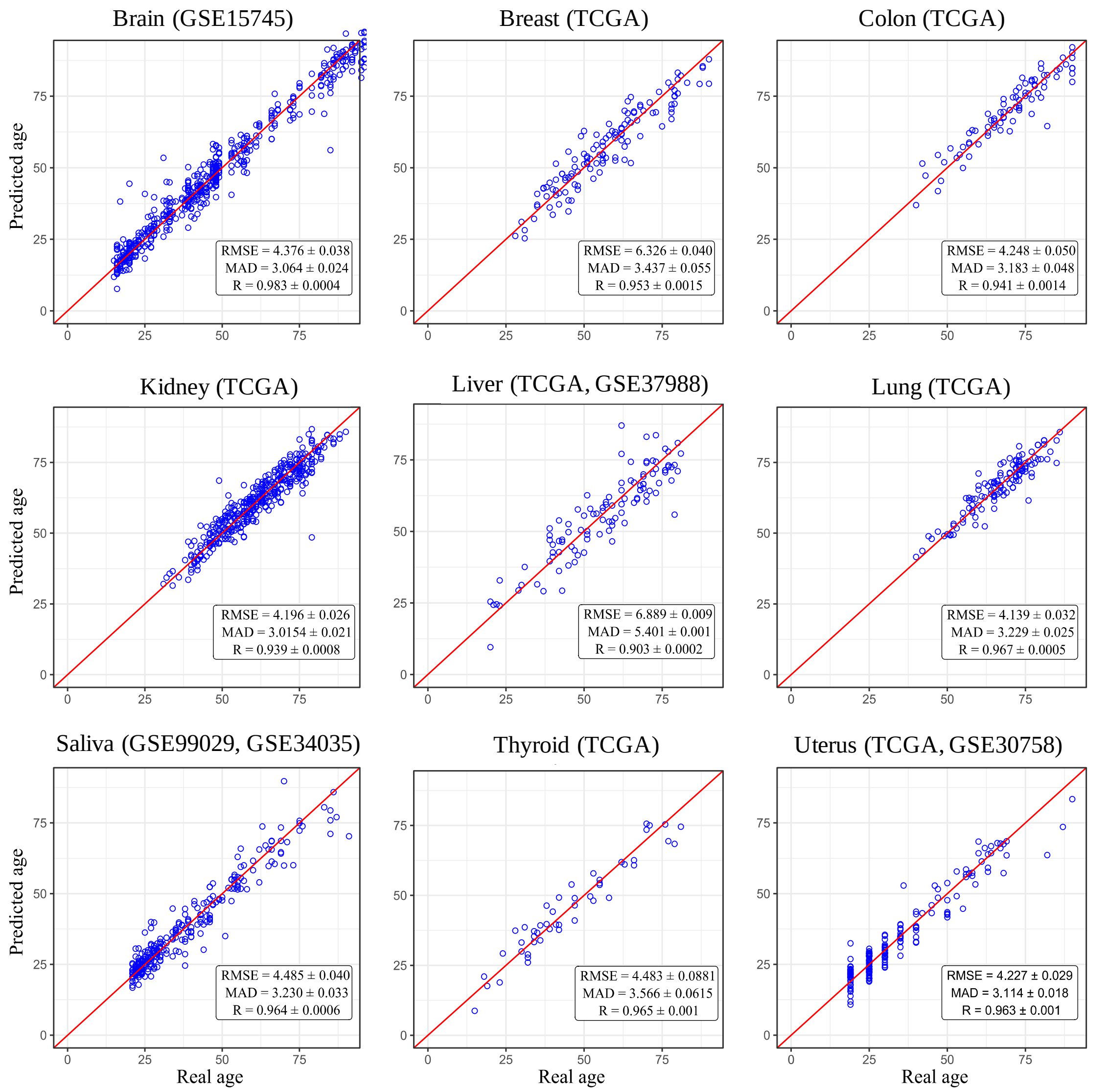

Supplementary Figure 2. Internal validation. This is the results of 10-fold cross validation of proposed tissue-specific age prediction model. It shows MAD of 3.519 years and R of 0.961 on average in nine tissue models. Results in brain (MAD = 3.064, R = 0.983), breast (MAD = 3.437, R = 0.953), colon (MAD = 3.183, R = 0.941), kidney (MAD = 3.015, R = 0.939), liver (MAD = 5.401, R = 0.903), lung (MAD = 3.229, R = 0.967), saliva (MAD = 3.230, R = 0.964), thyroid (MAD = 3.566, R = 0.965), uterus (MAD = 3.114, R = 0.963) are shown.

(a)

(b)

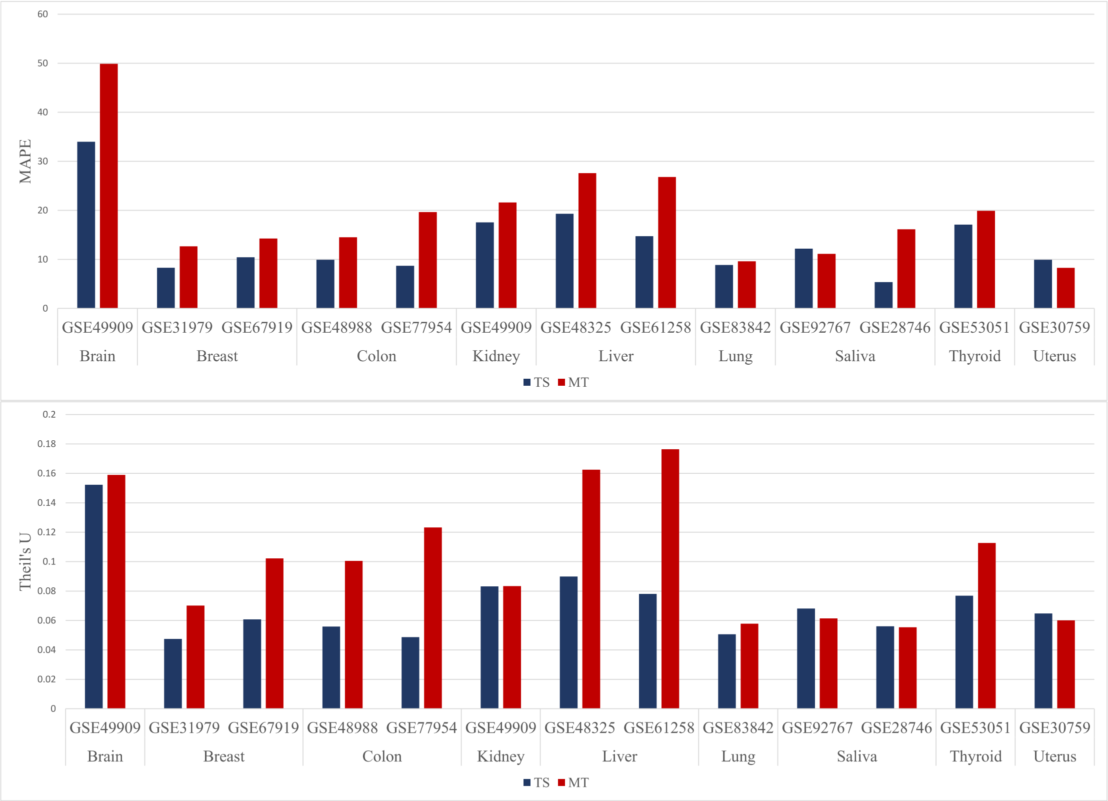

Supplementary Figure 3. Comparison with other metric. When we compared the performance of proposed tissue-specific age predictors and multi-tissue age predictors using other regression metrics, tissue-specific age predictors also showed better performance. (a) This is the result of Mean Absolute Percentage Error (MAPE) of each model in independent datasets from nine tissues. In almost all dataset, tissue-specific age predictors showed lower MAPE. (b) This is the result of Theil's U statistics of each model in independent datasets from nine tissues. Likewise, tissue-specific age predictors showed lower Theil's U statistics.

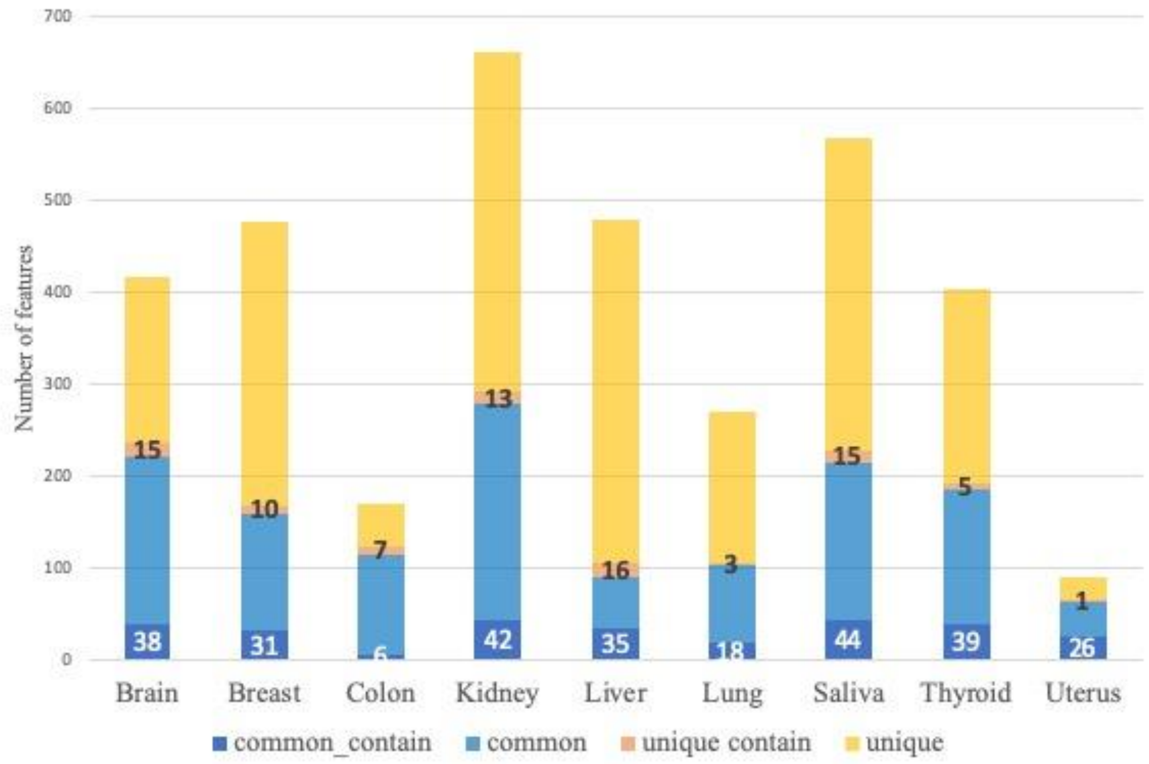

Supplementary Figure 4. Number of common features with hovarth. This figures showed the number of common features with features used for multi-tissue age predictors. In every 9 tissues, less than 10% of features are overlapped with multi-tissue age predictors. It shows that many features are uniquely attributed to tissue-specific age prediction. Besides, tissue-common groups more contain features of multi-tissue age predictor.

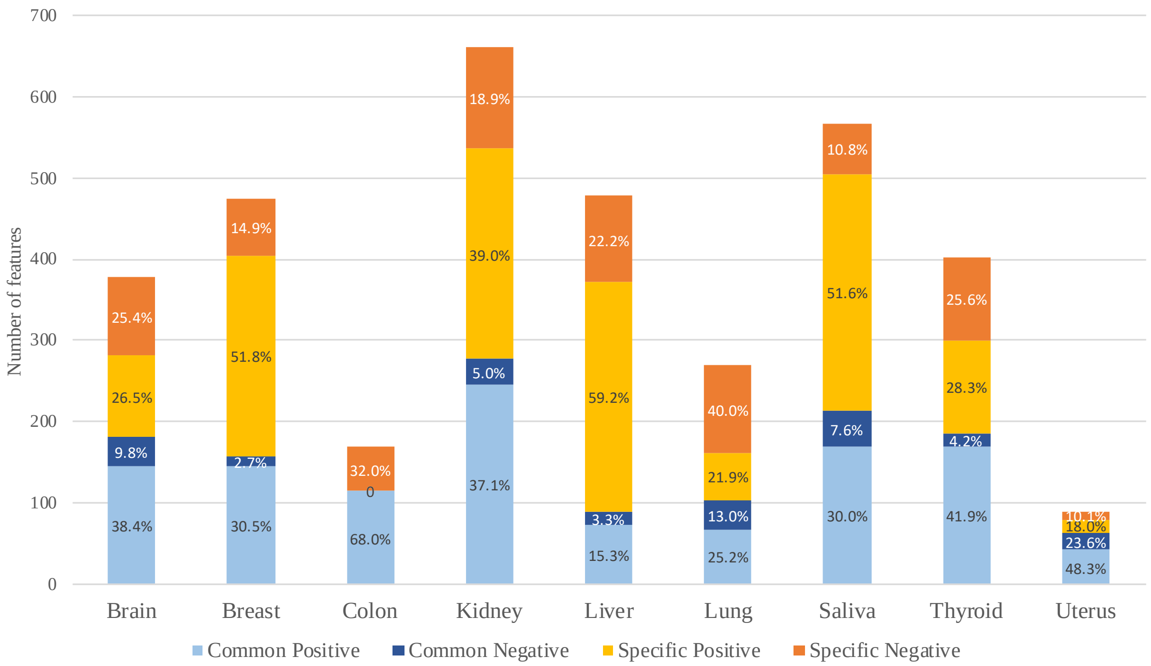

Supplementary Figure 5. Ratio of positive and negative ageCGS This figures showed the ratio of positive ageCGs and negative ageCGs in tissue-common and tissue-specific groups. Generally, more positive ageCGs were found, but the tissue-specific group had more negative ageCGs than that of tissue-common group.

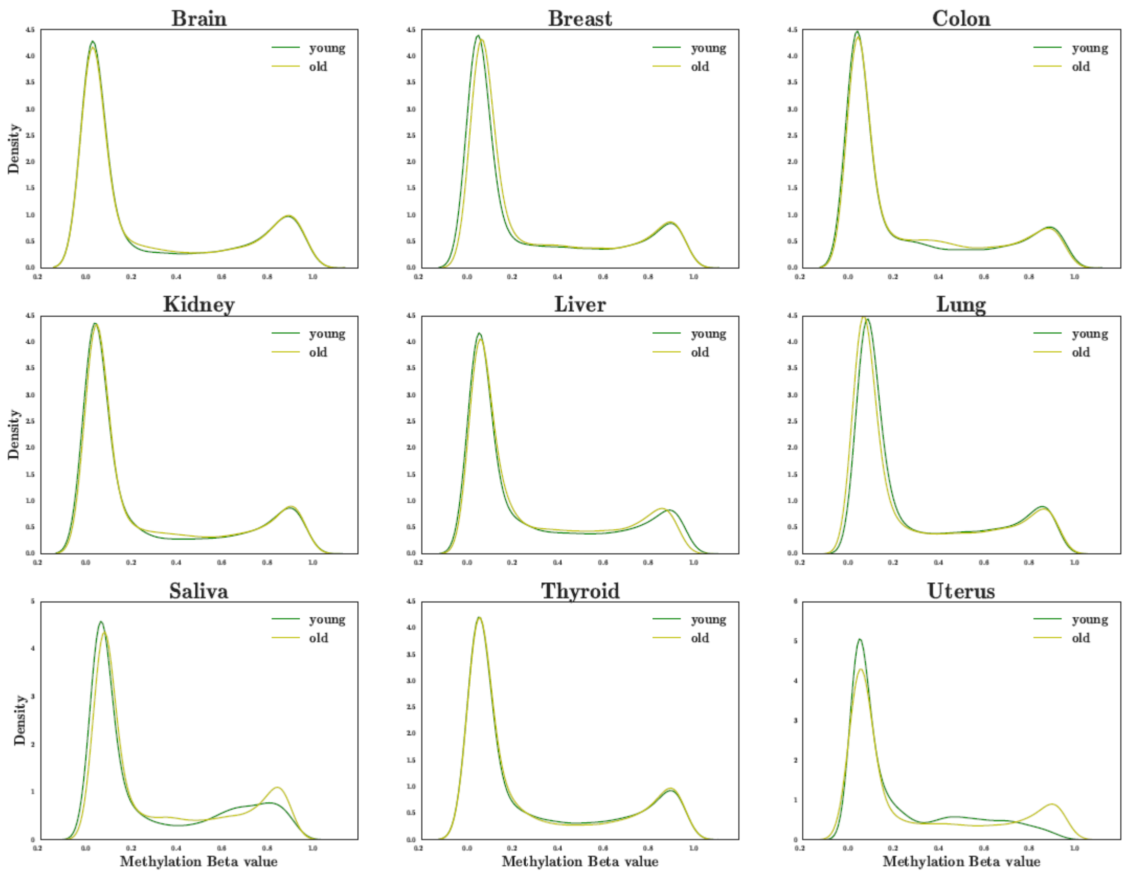

Supplementary Figure 6a. General methylation pattern difference in young and old group. These figures showed total methylation difference in young and old group of each tissue dataset. We compared the distribution of beta-value of whole markers in 27K platform. As a result, there are no statistically significant differences in two groups when we compared every methylation site.

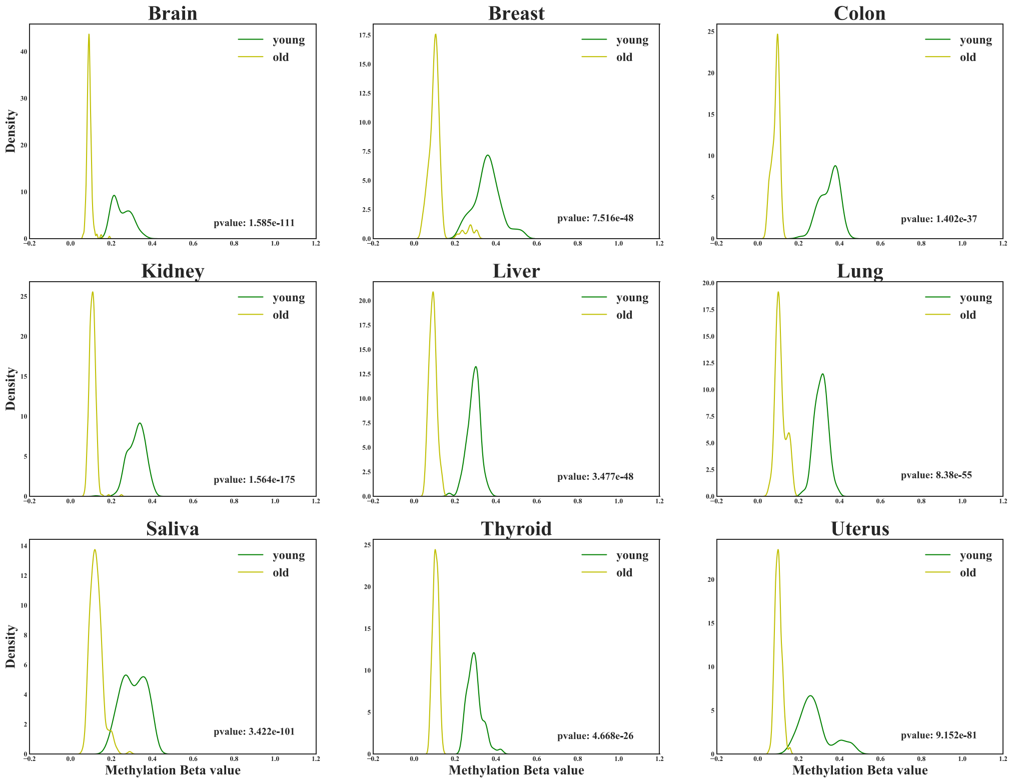

Supplementary Figure 6b. Aging-related methylation pattern difference in young and old group. These figures show that aging-related methylation sites has clear difference in old group than those of young group. Generally, Aging-related CpG sites are more methylated by getting aged. When we compared the distribution of beta-value of selected features in each tissue model, two groups showed significant differences(kolmogorov smirnov test).

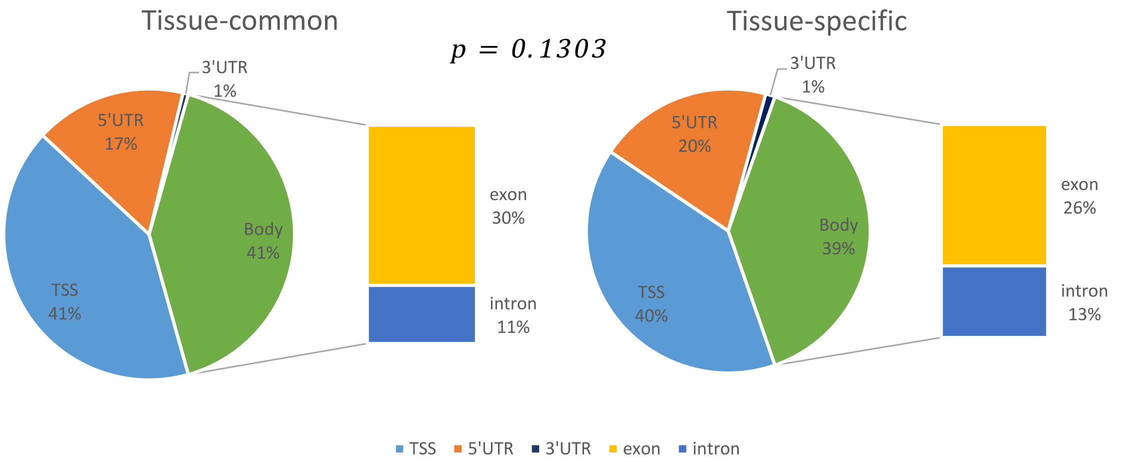

Supplementary Figure 7. Exon and intron ratio in tissue-common and tissue-specific group. This is the ratio of methylation regions in gene body regions in tissue-common and specific group. Although it is not significant, exon and intron ratio of two groups show slightly different ratio.

pos/neg	GOID	GOTerm	PValue	Corrected Pvalue
Positive ageCGs	GO:0007156	homophilic cell adhesion via plasma membrane adhesion molecules	0.00000	0.00002
	GO:0060452	positive regulation of cardiac muscle contraction	0.00001	0.00009
	GO:0090659	walking behavior	0.00002	0.00016
	GO:0045989	positive regulation of striated muscle contraction	0.00003	0.00023
	GO:0051931	regulation of sensory perception	0.00051	0.00191
Negative ageCGs	GO:0050795	regulation of behavior	0.00182	0.00182
	GO:0035914	skeletal muscle cell differentiation	0.00175	0.00219
	GO:0009301	snRNA transcription	0.00175	0.00219
	GO:0042795	snRNA transcription from RNA polymerase II promoter	0.00175	0.00219
	GO:0050434	positive regulation of viral transcription	0.00044	0.00222

Supplementary Table 4. Gene Ontology Analysis of tissue-common group. This is the top five significant gene ontology in positive and negative ageCGs in tissue-common group. Corrected p-value is calculated using Benjamini-Hochberg methods.

Tissue	GOID	GOTerm	PValue	Corrected Pvalue
Brain	GO:0001755	neural crest cell migration	0.00048	0.00241
	GO:0007422	peripheral nervous system development	0.00245	0.00307
Breast	GO:0033146	regulation of intracellular estrogen receptor signaling pathway	0.00192	0.01306
	GO:0061377	mammary gland lobule development	0.00584	0.01656
Colon	GO:0030513	positive regulation of BMP signaling pathway	0.00005	0.00070
	GO:0016114	terpenoid biosynthetic process	0.00033	0.00094
Kidney	GO:0071300	cellular response to retinoic acid	0.00177	0.00530
	GO:0048384	retinoic acid receptor signaling pathway	0.00159	0.00794
Liver	GO:0021591	ventricular system development	0.00002	0.00058
	GO:2000738	positive regulation of stem cell differentiation	0.00023	0.00145
Lung	GO:0090314	positive regulation of protein targeting to membrane	0.00002	0.00003
	GO:0090313	regulation of protein targeting to membrane	0.00003	0.00003
Saliva	GO:0003197	endocardial cushion development	0.00001	0.00020
	GO:0034332	adherens junction organization	0.00007	0.00063
Thyroid	GO:0048644	muscle organ morphogenesis	0.00304	0.00390
	GO:0043525	positive regulation of neuron apoptotic process	0.00088	0.00396

Supplementary Table 5a. Gene Ontology Analysis of positive ageCGs of tissue-specific group. This is the result of top two significant gene ontology in positive ageCGs in tissue-specific group in each tissue. Because uterus model had too small number of features for analysis, it was excluded from gene ontology analysis table.

Tissue	GOID	GOTerm	PValue	Corrected Pvalue
Brain	GO:0048640	negative regulation of developmental growth	0.00023	0.00301
	GO:0071850	mitotic cell cycle arrest	0.00166	0.00720
Breast	GO:1900077	negative regulation of cellular response to insulin stimulus	0.00003	0.00015
	GO:0046627	negative regulation of insulin receptor signaling pathway	0.00002	0.00024
Kidney	GO:0002576	platelet degranulation	0.00016	0.00096
	GO:0007595	lactation	0.00118	0.00236
Liver	GO:2000404	regulation of T cell migration	0.00006	0.00025
	GO:2000401	regulation of lymphocyte migration	0.00020	0.00039
Lung	GO:0050654	chondroitin sulfate proteoglycan metabolic process	0.00028	0.00103
	GO:0019800	peptide cross-linking via chondroitin 4-sulfate glycosaminoglycan	0.00013	0.00142
Saliva	GO:0051447	negative regulation of meiotic cell cycle	0.00072	0.00145
	GO:0051445	regulation of meiotic cell cycle	0.00312	0.00312
Thyroid	GO:0097006	regulation of plasma lipoprotein particle levels	0.00018	0.00036
	GO:0034381	plasma lipoprotein particle clearance	0.00068	0.00068

Supplementary Table 5b. Gene Ontology Analysis of negative ageCGs of tissue-specific group. This is the result of top two significant gene ontology in negative ageCGs in tissue-specific group in each tissue. Because uterus and colon had too small number of features for analysis, they were excluded from gene ontology analysis table.

cg22736354	Brain	Breast	Colon	Kidney	Liver	Lung	Saliva	Thyroid	Uterus
**Slope**	267.78	116.8	55.08	83.83	134.29	55.04	147.27	235.4	150.8
**Correlation(R)**	0.89	0.64	0.36	0.53	0.68	0.33	0.71	0.65	0.83
**F-test**	<0.05	<0.05	<0.05	<0.05	<0.05	<0.05	<0.05	<0.05	<0.05

Supplementary Table 6. **The comparison with cg22736354 methylation and age in each tissue.** we noted that the slopes of the methylation level of this single cg22736354 were calculated by single linear fit model with age. R represents Pearson's correlation coefficient. F-test represents the values in table are less than FDR 0.05.

References

Ahrens, M., O. Ammerpohl, W. von Schönfels, J. Kolarova, S. Bens, T. Itzel, A. Teufel, A. Herrmann, M. Brosch and H. Hinrichsen (2013). "DNA methylation analysis in nonalcoholic fatty liver disease suggests distinct disease-specific and remodeling signatures after bariatric surgery." Cell metabolism **18**(2): 296-302.

Bocklandt, S., W. Lin, M. E. Sehl, F. J. Sánchez, J. S. Sinsheimer, S. Horvath and E. Vilain (2011). "Epigenetic predictor of age." PloS one **6**(6): e14821.

Day, K., L. L. Waite, A. Thalacker-Mercer, A. West, M. M. Bamman, J. D. Brooks, R. M. Myers and D. Absher (2013). "Differential DNA methylation with age displays both common and dynamic features across human tissues that are influenced by CpG landscape." Genome biology **14**(9): R102.

Hair, B. Y., Z. Xu, E. L. Kirk, S. Harlid, R. Sandhu, W. R. Robinson, M. C. Wu, A. F. Olshan, K. Conway and J. A. Taylor (2015). "Body mass index associated with genome-wide methylation in breast tissue." Breast cancer research and treatment **151**(2): 453-463.

Hong, S. R., S.-E. Jung, E. H. Lee, K.-J. Shin, W. I. Yang and H. Y. Lee (2017). "DNA methylation-based age prediction from saliva: high age predictability by combination of 7 CpG markers." Forensic Science International: Genetics **29**: 118-125.

Horvath, S. (2013). "DNA methylation age of human tissues and cell types." Genome Biol **14**(10): R115.

Horvath, S., W. Erhart, M. Brosch, O. Ammerpohl, W. von Schönfels, M. Ahrens, N. Heits, J. T. Bell, P.-C. Tsai and T. D. Spector (2014). "Obesity accelerates epigenetic aging of human liver." Proceedings of the National Academy of Sciences **111**(43): 15538-15543.

Kajiura, K., K. Masuda, T. Naruto, T. Kohmoto, M. Watabnabe, M. Tsuboi, H. Takizawa, K. Kondo, A. Tangoku and I. Imoto (2017). "Frequent silencing of the candidate tumor suppressor TRIM58 by promoter methylation in early-stage lung adenocarcinoma." Oncotarget **8**(2): 2890.

Noreen, F., M. Röösli, P. Gaj, J. Pietrzak, S. Weis, P. Urfer, J. Regula, P. Schär and K. Truninger (2014). "Modulation of age-and cancer-associated DNA methylation change in the healthy colon by aspirin and lifestyle." JNCI: Journal of the National Cancer Institute **106**(7).

Qu, X., T. Sandmann, H. Frierson Jr, L. Fu, E. Fuentes, K. Walter, K. Okrah, C. Rumpel, C. Moskaluk and S. Lu (2016). "Integrated genomic analysis of colorectal cancer progression reveals activation of EGFR through demethylation of the EREG promoter." Oncogene **35**(50): 6403.

Timp, W., H. C. Bravo, O. G. McDonald, M. Goggins, C. Umbricht, M. Zeiger, A. P. Feinberg and R. A. Irizarry (2014). "Large hypomethylated blocks as a universal defining epigenetic alteration in human solid tumors." Genome medicine **6**(8): 61.

Zhuang, J., A. Jones, S.-H. Lee, E. Ng, H. Fiegl, M. Zikan, D. Cibula, A. Sargent, H. B. Salvesen and I. J. Jacobs (2012). "The dynamics and prognostic potential of DNA methylation changes at stem cell gene loci in women's cancer." PLoS genetics **8**(2): e1002517.
